# Potential Risk Factors for the Onset of Complex Regional Pain Syndrome Type 1: A Systematic Literature Review

**DOI:** 10.1155/2015/956539

**Published:** 2015-01-26

**Authors:** Tracey Pons, Edward A. Shipton, Jonathan Williman, Roger T. Mulder

**Affiliations:** ^1^Department of Anaesthesia, University of Otago, Christchurch, Corner of Riccarton and Hagley Avenues, Christchurch 8042, New Zealand; ^2^Department of Population Health, University of Otago, Christchurch, Corner of Riccarton and Hagley Avenues, Christchurch 8042, New Zealand; ^3^Department of Psychological Medicine, University of Otago, Christchurch, Corner of Riccarton and Hagley Avenues, Christchurch 8042, New Zealand

## Abstract

Anaesthetists in the acute and chronic pain teams are often involved in treating Complex Regional Pain Syndromes. Current literature about the risk factors for the onset of Complex Regional Pain Syndrome Type 1 (CRPS 1) remains sparse. This syndrome has a low prevalence, a highly variable presentation, and no gold standard for diagnosis. In the research setting, the pathogenesis of the syndrome continues to be elusive. There is a growing body of literature that addresses efficacy of a wide range of interventions as well as the likely mechanisms that contribute to the onset of CRPS 1. The objective for this systematic search of the literature focuses on determining the potential risk factors for the onset of CRPS 1. Eligible articles were analysed, dated 1996 to April 2014, and potential risk factors for the onset of CRPS 1 were identified from 10 prospective and 6 retrospective studies. Potential risk factors for the onset of CRPS 1 were found to include being female, particularly postmenopausal female, ankle dislocation or intra-articular fracture, immobilisation, and a report of higher than usual levels of pain in the early phases of trauma. It is not possible to draw definite conclusions as this evidence is heterogeneous and of mixed quality, relevance, and weighting strength against bias and has not been confirmed across multiple trials or in homogenous studies.

## 1. Introduction

There is a growing body of literature addressing a variety of disorders known as Complex Regional Pain Syndrome (CRPS). It is a condition that presents with a pain experience that is severe and disproportionate to the inciting event and is accompanied by highly variable signs and symptoms of inflammatory, sensory, autonomic, trophic, or motor features. Anaesthetists in the acute and chronic pain teams are often involved in treating Complex Regional Pain Syndromes. The onset of CRPS can follow injuries ranging from minor injuries to fracture(s), from lesions of the central nervous system, or from surgery [1–3]. Its prevalence is low, ranging from 5.46 to 26.2 per 100 000 [4, 5]. This low prevalence has led to difficulty in research where robust statistical analysis necessitates larger sample sizes [6, 7].

Furthermore, CRPS nomenclature continues to be debated and remains controversial [8–11]. Research had shown that this condition is not wholly a problem of the sympathetic nervous system. The old terms “reflex sympathetic dystrophy” and “causalgia” needed to be changed [12]. In 1994, the committee for taxonomy of the International Association for the Study of Pain (IASP) identified specific diagnostic criteria for this syndrome that were termed the IASP criteria. This IASP committee changed the name to Complex Regional Pain Syndrome or CRPS. The term CRPS Type 1 (CRPS 1) applies, if there is no nerve damage, or CRPS Type 11, if the nerve is physically and permanently damaged, and this nomenclature replaced the terms “reflex sympathetic dystrophy” and “causalgia,” respectively [13]. Other diagnostic criteria developed were the Veldman [14] and Harden/Bruehl [15] criteria that continue to be used in clinical practice and research. The Harden/Bruehl criteria became known as “The Budapest Criteria” with minor modifications. Though published in an IASP-sanctioned book, the Harden/Bruehl criteria have not been officially endorsed by the IASP. The “Budapest Criteria” are used in clinical diagnosis. Here a report of at least one symptom in 3 or 4 categories (sensory, vasomotor, sudomotor/oedema, motor/trophic) with at least one sign at time of evaluation in 2 or more of the categories (sensory, vasomotor, sudomotor/oedema, motor/trophic) confirms a clinical CRPS diagnosis. There must be no other diagnosis that better explains the signs and symptoms. Budapest Clinical Criteria have retained sensitivity almost identical to the IASP criteria but with much improved specificity.

In the “Budapest Research Criteria,” diagnostic decision rule is at least one symptom in all four symptom categories and at least one sign (observed at evaluation) in two or more sign categories. The intent of the Budapest Research Criteria was to maximize specificity (minimize false positives) at the expense of sensitivity. They have a high specificity but a low sensitivity [16, 17]. This systematic review explores the literature since 1999. It therefore includes a level of variation for diagnostic criteria.

CRPS 1 is considered by most to be overdiagnosed [6, 18, 19]. There are a few, however, who still consider it underdiagnosed [20]. The precise pathophysiological mechanisms and predictive factors underlying CRPS 1 or subsets of CRPS 1 remain unknown [6, 21–23]. A standard diagnostic test is unavailable and the absence of a gold standard makes the validation of diagnostic criteria difficult [6, 24, 25]. Effective treatment strategies (in both the research and clinical fields) have moderate evidence [26–29]. A variety of medical and physiotherapy interventions and a multidisciplinary approach to the management of CRPS 1 continue to be widely used [23, 30, 31]. These factors contribute to the difficulty in determining potential risk factors for CRPS 1 in a reliable and statistically valid way.

There has been no systematic review of risk factors which may contribute to the onset of CRPS 1. This is the first systematic review to address factors posing as possible risk factors for the onset of CRPS 1. This paper selects from the current literature to systematically describe factors which expose a potential risk factor for a possible relationship to the onset of CRPS 1. This paper defines a risk factor as a factor contributing to a likely association of the onset of CRPS 1. This association is not necessarily causal.

## 2. Materials and Methods

### 2.1. Study Selection

Key words for CRPS (such as diagnosis, epidemiology, aetiology, genetics, history, pathophysiology, rehabilitation, risks, fractures, osteoporosis, or predictors) were combined in searches of Web of Science and OVID Medline for articles dated 1996 to April 2014. All abstracts were screened. Inclusion criteria for data extraction were articles written in English with reference to risks or predictors associated with the onset of CRPS 1. Exclusion criteria included articles written in other languages or no mention of CRPS 1 risks for either onset or outcomes or prognosis. A total of 969 abstracts were screened according to the study selection inclusion and exclusion criteria. Forty-one abstracts were included, and 928 abstracts were excluded. Search terms are outlined in Table 1 (Web of Science search) and Table 2 (Groups of OVID Medline searches).

### 2.2. Data Extraction

**Figure 1:**
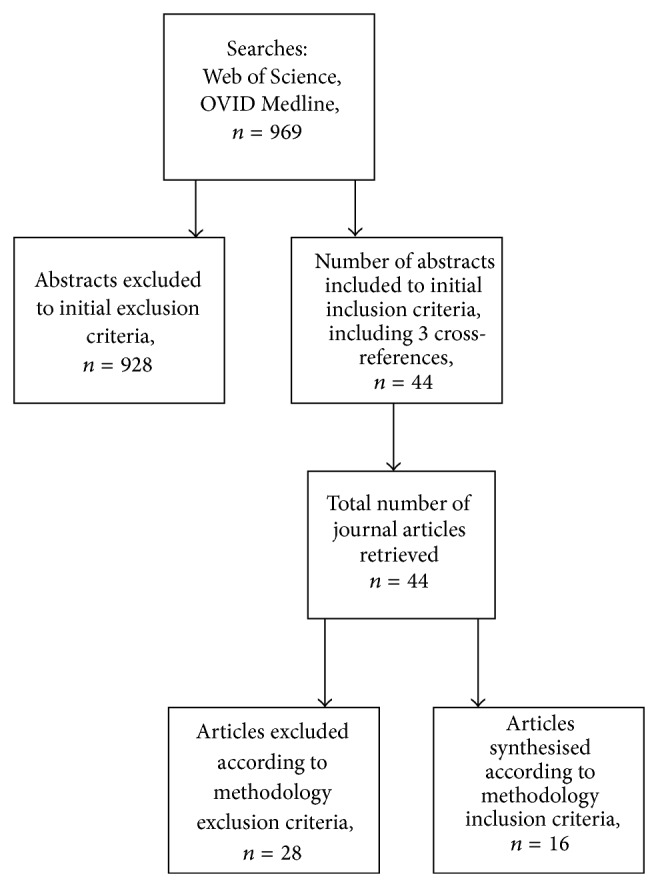
Summary of data extraction.

The methodology of the 41 articles included through the study selection was screened for data extraction with these inclusion criteria being randomised controlled trials, prospective and retrospective studies for CRPS 1. Exclusion criteria incorporated CRPS Type 11, methodology used in animal studies, case studies, and case control studies or cross-sectional studies. Sixteen articles met these inclusion criteria (10 prospective studies, 6 retrospective studies). Twenty-five articles were excluded due to the direction of effect not being able to be determined. These inclusion and exclusion criteria were used to determine evidence for a direction of the effect specific for the likelihood or not for the potential risk for the onset of CRPS 1. A prospective or retrospective study can provide evidence for the likelihood (or not) of a risk towards the onset of a disease by determining a direction of effect. Animal studies, case studies, case control studies, and cross-sectional studies provide evidence of a relationship. They are not able to determine the direction of effect of this relationship over a period of time. Longitudinal, prospective, or retrospective studies by nature of their design are more able to determine this necessary direction of effect to reveal the potential risks for the onset of disease [7].  Figure 1 summarises the data extraction.

## 3. Results

### 3.1. Data Synthesis

No randomised controlled trials (RCTs) were found describing either possible risk or predictive factors for the onset of CRPS 1. Ten prospective studies and six retrospective studies (total of 16) were included for the data synthesis. These data were synthesised from the following patient samples: 77 patients after knee replacement surgery; 1976 patients after distal fractures of the radius; 748 patients after wrist or ankle fractures; 168 CRPS male patients from the Turkish armed forces; 1639 CRPS 1 male and female patients with duration of disease for <1 year; and 453 male and female CRPS 1 patients with duration of disease >1 year. Four hundred and sixty patients were lost to follow-up; 21 eligible fracture patients were lost due to administrative errors; 216 patients refused to participate; there were 1052 controls (male and female). The human populations from which the patient epidemiological studies were sourced consisted of a total of 297,372 people. Eight studies used the IASP criteria for diagnosis. Six studies used a variation between the Veldman and Harden/Bruehl criteria. One study did not define their criteria stating their use of “standard criteria.” Criteria were not stated at all in one study. In the prospective studies, follow-up time varied from 3 months to 2 years. In the retrospective studies, follow-up time varied from 3 to 10 years.  Table 3 describes the data from the literature synthesised from prospective studies.  Table 4 describes the data from the literature synthesised from retrospective studies.

### 3.2. Data Analysis

The data show a high level of heterogeneity. There are no particular variables consistent across these studies with evidence strong enough to comprise a risk factor. Rating criteria for quality and relevance and weighted strength against bias were based on published recommendations [7, 62, 63]. Quality and relevance criteria used included the following: the sample had to be representative of the CRPS 1 population; an adequate control group was needed; study attrition rate was required; adequate description of study and measurements used were necessary (to identify a potential risk factor); the statistical analysis needed to be appropriate.

The data were analysed for a weighted strength against the risk of possible bias. The criteria used included bias risk in sample selection, study design, funding provision, detection, and measurement. Two authors (Tracey Pons, Roger T. Mulder) independently assessed each paper for quality, relevance, and weighted strength against potential bias. Any disagreement was discussed and resolved by consensus. Where a disagreement could not be resolved by consensus, the two other authors (Edward A. Shipton, Jonathan Williman) arbitrated disagreement and facilitated consensus amongst all four authors. Observer expectancy was considered to be reduced since 2 authors' (Roger T. Mulder, Jonathan Williman) expertise is outside the pain management field; hence, no external observer was included as they were considered objective enough with no historical or current involvement with CRPS 1 diagnosis or management.

Quality and relevance were measured against six factors. Ratings were graded as poor, adequate, or good with the following algorithm: good = five or six factors rated as yes; adequate = three or four factors rated as yes; and poor = less than two factors rated as yes. The weighting against bias was measured against five factors. Ratings were graded as weak, acceptable, or strong with the following algorithm: strong = all five factors rated as no risk; acceptable = three or four factors rated as no risk; and weak = two or less factors rated as no risk.

These data provide a broad and heterogeneous research platform towards probing for possible risk factors for the onset of CRPS 1. In this systematic review, 2 prospective studies and 3 retrospective studies were rated as good (total of 5). Four prospective studies and 1 retrospective study were rated as adequate (total of 5). Four prospective studies and 2 retrospective studies were rated as poor (total of 6). For the weighted strength against bias, 7 prospective studies were weak, 2 were acceptable, and 1 was strong. In the retrospective studies, 3 were weak, 2 were acceptable, and 1 was strong. In summary for the weighted strength against bias, 10 were weak, 4 were acceptable, and 2 were strong. However, these findings should be treated with caution as their statistical reliability and consistency have not been established across multiple or homogeneous studies.

The quality and relevance data are outlined in Table 5 for prospective studies and in Table 6 for retrospective studies. The weighting strength against bias data is illustrated in Table 7 for prospective studies and in Table 8 for retrospective studies.

The following are shown* not* to be risk factors for the onset of CRPS 1: namely, preoperative psychological distress; preoperative pain levels (with poor quality, poor relevance, and weak weighting against bias); psychological behaviour and depression (with adequate quality and relevance but with weak weighting against bias); and a diagnostic bone scan (with adequate quality and relevance but with weak weighting against bias). The factors not considered to be risk factors for the onset of CRPS 1 are summarised in Table 9.

The potential risk factors identified with a strong weighting against bias as well as good quality and relevance are being female (particularly postmenopausal), a fracture of the distal radius, and dislocation or an intra-articular fracture of the ankle. The factors presenting as possible risks for the onset of CRPS 1 are summarised in Table 10.

## 4. Discussion

Potential risk factors identified (strong weighting against bias, good quality, and relevance) across the 16 papers are as follows: being female (particularly postmenopausal); obtaining a fracture of the distal radius; suffering an ankle dislocation or intra-articular fracture; and reports of higher than usual levels of pain in the early phases after trauma. The findings of this systematic review should be treated with caution as their statistical reliability and consistency have not yet been established across multiple or homogeneous studies and diagnostic criteria were mixed with Budapest Criteria not being used.

Age was accounted for in most studies. Age as a consistent potential risk factor for the onset of CRPS 1 could not be identified. This is shown in the population studies by Sandroni et al. [4], Moseley et al. [41], and de Mos et al. [5], as well as in the study by van Rijn et al. [22]. Although most of these data show that the risk increases in postmenopausal women [5, 34–38, 40], the retrospective studies by Allen et al. [42] and by Anderson and Fallat [44] show a lower age. This might be due to average age of the group sample with the inclusion of both genders. The average age of the female sample groups is, unfortunately, not provided in either of these studies. Females at any age pose a higher risk for the onset of CRPS 1. However, the study of males in the armed forces by Duman et al. [43] shows that males are vulnerable as well. This systematic review shows that the onset of CRPS Type 1 is higher in females than in males in the mixed gender studies.

The data show the cause of the inciting event to be mixed. It can be related to surgery, fractures, or soft tissue injuries. The presence of other comorbidities is neither predictive nor a risk factor for the onset of CRPS 1. Reports of higher than usual levels of pain in early phase of trauma were cited as strong evidence of a risk factor by Beerthuizen et al. [35] but as weak evidence by Jellad et al. [38].

Psychosocial factors are weakly weighted as a risk factor for the onset of CRPS 1. Moseley et al. find catastrophising not to be predictive for the onset of CRPS [41]. This is confirmed by other reviews and studies investigating psychological influences on the onset and progression of CRPS [58, 59, 64–66]. Psychological behaviour, depression, and preoperative psychological distress or pain levels are not predictive of the onset of CRPS. The earlier literature described the “Sudeck A personality,” a personality of high anxiety, as a likely risk factor towards the onset of CRPS 1 [61]. A high anxiety personality trait was identified by this systematic review as only a weak potential risk [37]. In other persistent pain conditions, these complex interactions between the onset of the pathogenesis of CRPS 1 and psychological factors are predictive of level of function [45, 60]. Their interaction in CRPS 1 continues to be investigated by clinicians and researchers [5, 64].

These data show that a positive diagnostic bone scan is not a risk factor for the onset of CRPS 1 [46] and has been confirmed by other studies [42, 47, 48]. Interobserver consistency with interpretation of bone scans appears to be variable [49]. However, a diagnostic bone scan has been found to be helpful towards a diagnosis of CRPS 1 in some observations [50, 51], but since recovery of bone mass following ankle fractures remains variable, it is not necessarily indicative of CRPS 1 [52].

Two recent systematic reviews have collated the prognostic findings about CRPS 1 [53, 67]. Both agreed that the quality of evidence is poor. Our systematic review has confirmed this regarding the risks of onset of CRPS 1. One review retrieved 1648 relevant papers of which twelve were robust enough for qualitative analysis [53]. Prognostic factors for poor outcomes were grouped within 7 clinical clusters as follows: (1) gender, where two studies show the male gender and one study shows the female gender; (2) age, where there is a high variation in age of onset affecting prognosis; (3) inciting event, such as polytrauma, inciting event other than fracture, severe initial injury, and distal articular location; (4) localisation site, either upper or lower extremity; (5) clinical features, such as exercised induced pain, sensory disturbances, initial cold skin temperature, complications of infection, skin ulcers, chronic oedema, dystonia or myoclonus, algodystrophy score > 7 out to 10, low SF-36 general health score, disease duration > 1 year, and coexistence of misdiagnosed nerve injury and compression; (6) associated comorbidities included alcoholism and psychological background in nontraumatic CRPS 1; and (7) diagnosis where a delay of >2 months after inciting event was shown to be associated with poorer outcomes. The other review found that many CRPS 1 patients recover in 6–13 months but that a significant number continue to experience persistent pain and disability [67].

### 4.1. Implications for Research

This systematic review highlights potential risk factors that will contribute to future exploration about the onset of CRPS 1. Identifying risk factors associated with a poor prognosis is important as well. Risk factors for the onset of CRPS 1 identified in this systematic review may or may not be associated with a poor prognosis. The low prevalence of CRPS 1, its heterogeneous presentation, and its lack of highly specific or sensitive diagnostic criteria as well as the lack of clarity around consensus for these criteria create challenges in carrying out research [4, 6, 21, 42, 54, 55]. In CRPS 1, more trials across different settings are needed. The cross-sectional and case control studies excluded from this systematic review may still offer insight into the development of future longitudinal studies to determine direction and strength of the effects.

### 4.2. Implications for Clinical Practice

No specific or sensitive clinical sign or clinical symptom was shown in this review to pose a risk factor for the onset of CRPS 1. Clarity around the sensitivity and specificity of laboratory and imaging testing is needed [42, 47, 48, 51, 52]. This review confirms the importance of maintaining clinician awareness and of being aware of potential risk factors to enable the early diagnosis of CRPS 1 [56]. Evaluation by experienced clinicians hastens the diagnosis [36, 57]. Early diagnosis and referral to pain management specialists and physiotherapists are related to better outcomes [53, 68, 69]. Moseley et al. [41] suggested that a pain score of ≥5 in the first week of fracture could be considered a “red flag” risk for the likely onset of CRPS 1.

## 5. Conclusion

This systematic review shows that the accurate potential risk factors for the onset of CRPS 1 remain elusive. Studies remain heterogeneous, of mixed quality and relevance, and with varied weighting against the risks of bias. The low prevalence of CRPS 1 accompanied by a lack of a gold standard for diagnosis contributes to the difficulties around determining potential risk factors for the onset of CRPS 1.

Potential risk factors identified with strong weighting against bias and good quality and relevance are summarised as follows: being female (particularly postmenopausal); obtaining a fracture of the distal radius; suffering an ankle dislocation or intra-articular fracture; and reports of higher than usual levels of pain in the early phases after trauma. Potential risk factors with much weaker weighting against bias and poorer quality and relevance include immobilisation, psychosocial barriers, and a positive diagnostic bone scan. Definite conclusions cannot be drawn as evidence remains inconsistent across multiple trials or in homogenous studies.

## Figures and Tables

**Table 1:** Search terms for Web of Science CRPS 1 risks.

Database	Search statement (1996 to April 2014)	Results
Web of Science	CRPS and risks	128

**Table 2:** Search terms for OVID Medline(R) CRPS 1 risks.

Number	Search statement	Results
OVID Medline(R) < 1996 to April 2014		
1	Complex Regional Pain Syndromes (diagnosis, epidemiology, aetiology, genetics, history, physiopathology, and rehabilitation)	617

OVID Medline(R) < 1996 to April 2014		
1	^*^Complex Regional Pain Syndromes/ep, et (Epidemiology, Aetiology)	104
2	crps.tw.	1122
3	Complex Regional Pain Syndrome^*^.tw.	1515
4	1 or 2 or 3	1823
5	^*^Epidemiology/	2325
6	epidemiology.tw.	63875
7	aetiology.tw.	22487
8	etiology.tw.	75941
9	5 or 6 or 7 or 8	159810
10	^*^Risk Factors/	592
11	^*^Risk/	1573
12	risk^*^.tw.	990294
13	10 or 11 or 12	990691
14	4 and 9 and 13	9
15	4 and 9	80

OVID Medline(R) < 1996 to April 2014		
1	Complex Regional Pain Syndromes/or Reflex Sympathetic Dystrophy/	2054
2	Fractures, Bone/or Osteoporotic Fractures/or Ulna Fractures/or Tibial Fractures/or Radius Fractures	31463
3	1 and 2	117

^*^Before the word indicates focussing the subject heading. This means the results that have been retrieved have that subject heading as a major topic in the article, rather than something more minor.

^*^After the word refers to truncation. This means searching for all words have the same start, but different endings. In this case, for search 12 e.g. risk^*^.tw would look for risk, risks, risky, and anything else that starts with risk.

**Table 3:** Characteristics of the prospective data literature synthesized.

Author	Number of initial sample	Outcome measure listed in italics followed by instrument used	Result for risk towards the onset of CRPS 1	Diagnostic criteria used for CRPS 1 diagnosis	Number in sample lost to follow-up, declined to participate, or study attrition (%)	Follow-up period
Harden et al. 2003 [32]	77 patients for total knee replacement	*Pain intensity*: McGill Pain Questionnaire *Depression*: Beck Depressive Inventory *Anxiety*: trait form of the State Trait Anxiety Inventory	CRPS 1-like symptoms following total knee replacement were not predicted by preoperative psychological distress or pain levels	IASP	26 (33.7%)	6 months

Schürmann et al. 2000 [33]	27 distal radial fracture patients	*Oedema*: Likert scale *Active ROM*: Likert scale *Sympathetic function*: laser Doppler flowmetry, inspiratory gasp test, and contralateral cooling test Age or gender is NOT included in analyses	Failure of the sympathetic nervous system predicted those who developed CRPS 1 in the early stages of patients who had radial fractures and also possibly suffer from a systemic sympathetic dysfunction that is not limited to the affected limb	IASP	None	12 weeks

Puchalski and Zyluk 2005 [34]	121 distal radial fractures Population group: postmenopausal women, retired, or disability pensioners	*Personality traits*: Eysenck Personality Questionnaire *Depression*: Beck Depressive Inventory if <60 years of age or Yesavage's Geriatric Depression Scale *CRPS 1 severity*: Zyluk scoring system	In 62 patients with distal radial fractures, 18% developed CRPS 1 (8 females, one male) Their psychological behaviour patterns or depression did not differ with those who had not developed CRPS 1	Veldman and Zyluk CRPS 1 scoring system	59 (48.7%) refuse permission for psychological examination Of 62 patients included, 12 (19.4%) were lost to follow-up	20 months

Beerthuizen et al. 2012 [35]	748 wrist or ankle fractures	*Health related quality of life*: SF-36 survey *Medical fracture details*: type, location of fracture with type of fracture and treatment or number of weeks in plaster	Of 596 patients with wrist or ankle fractures, 7% developed CRPS 1; wrist or ankle fracture dislocation and intra-articular fracture contributed significantly to the likelihood of the development of CRPS 1; one year following the fracture, no CRPS 1 patient was pain-free; the highest majority of patients were females (73%); the highest incidence was between 61 and 70 years of age; early reporting of high levels of pain and other musculoskeletal comorbidities made the risk of CRPS 1 more likely	3 sets of criteria: Veldman, IASP, and Harden/Bruehl as well as confirmation with experienced clinician	152 (20.3%) decline consent 46 (18.6%) are lost to follow-up	1 year

Dijkstra et al. 2003 [36]	91 distal radius fractures	*Pain*: Visual Analogue Scale *Stressful events beforefracture*: Social Readjustment Rating Scale	Only one female patient (age 69 years) developed CRPS 1 after a follow-up of 88 patients	IASP	3 are lost to follow-up	1 year

Dilek et al. 2012 [37]	74 with distal radius fractures treated with closed reduction and plaster casts	*Psychological assessment*: Anxiety Sensitivity Index, Toronto Alexithymia Scale-20, State Trait Anxiety Inventory, and Beck Depression Inventory	In 50 patients, a high risk for developing CRPS 1 was found in those with a high anxiety personality trait score; of the 50 patients, 26% (13/50) developed CRPS 1; 34% of the females (age 62.38 ± 10.8) developed CRPS; 11% of the males developed CRPS 1	IASP	13 (17.6%) refuse permission for psychological examination; 4 (5%) are excluded due to needing surgery; 7 (9%) are lost to follow-up	16 months

Jellad et al. 2014 [38]	121 consecutive patients with fractures of the distal radius treated conservatively	*Pain*: Visual Analogue Scale *Active rangeof motion*: Goniometer and Kapandji distance *Hand and wrist function*: Patient Related Wrist Evaluation *Depression or Anxiety*: Arabic adaption of Hospital Anxiety and Depression scale *Quality of Life*: Arabic adaption of SF-36	CRPS 1 occurred in 32.2% of patients, mostly females (age 52.9. ± 13.2) [odds ratio 5.774 95% CI 1.391–23.966]; these also reported severe pain and impairment of quality of life where the CRPS 1 onset occurred in the third and fourth week after cast removal	Veldman	31 (25.6%) excluded as treated operatively or other problems	9 months

Goris et al. 2007 [39]	114 distal radius fractures 95 females, 19 males, mean age of 62 years (range 22–82 years)	*Medical fracture details*: type, location of fracture with type of fracture and treatment or number of weeks in plaster *Skin temperature*: infrared ear thermometer and ThermaCAM E2 infrared camera *Oedema*: custom made device for measuring accurate finger circumference *Active range of motion*: Goniometer *Skin colour*: subjective Likert scale *Grip strength*: dynamometer *Blood analysis*: venous blood samples for lactate and oxygen saturation	CRPS 1 onset was associated with an increased regional inflammatory score (sensitivity 100%, specificity 16%); it was not associated with raised inflammatory markers in the blood; age and gender not included in published analyses	IASP and Harden/Bruehl criteria	25 (21.9%) are lost to follow-up	1 year

Gradl and Schürmann 2005 [40]	10 CRPS patients, 4 males and 6 females, age 53–79 years with average age of 62 years	*Sympathetic function*: laser Doppler flowmetry, inspiratory gasp test, and contralateral cooling test	Dysfunction of the sympathetic nervous system evident in the early stage of CRPS 1 was measured in this German study; this dysfunction was transitory; it normalised over the course of the syndrome; the diagnosis of CRPS 1 was able to be made 46 to 72 days following an injury	Harden/Bruehl	None	3 months

Moseley et al. 2014 [41]	1549 near consecutive patients with radial fractures across 3 hospital out-patients	*Pain*: *NRS* *Reaction time*: seconds *Dysynchiria*: absent or present *Swelling*: affected thumb and first 3 fingers' circumference as a proportion of unaffected hand *Catastrophising*: Pain Catastrophising Scale	A pain score of ≥5 in the first week of fracture is shown to be predictive and should be considered a “red flag” risk for the likely onset of CRPS 1; 55 patients have developed CRPS 1 at evaluation 112 days after fracture; age and gender were not predictive of CRPS 1 onset	Referred to as “established criteria” without formal reference	21 likely CRPS 1 patients lost due to administrative error; 93.3.% of all fractures eligible for inclusion and 94.5% agree to participate; 97.2% contacted for follow-up; no numbers of patients given, only percentages	Sequential cohort over 2 years

**Table 4:** Characteristics of the retrospective data literature synthesized.

Author	Number of initial sample	Outcome measure listed other than age and gender	Risk factor towards the onset of CRPS 1	Diagnostic criteria used for CRPS 1 diagnosis	Period time for inspection
Allen et al. 1999 [42]	134 CRPS patients; 70% female, 30% male Mean group age at evaluation 41.8 (18–71) years	Inciting injury Location Job related occupation Legal and worker compensation issues Season Bone scans Treatment, medical and allied Immobilisation Myofascial component	A diagnostic bone scan was not predictive of a CRPS 1 diagnosis The inciting event was sprains in 29%, surgical procedures in 24%, and fractures in 16%; since physician imposed immobilisation in either a cast or splint involved 47% of the sample, the possibility of immobilisation is raised as a possible risk factor as well	IASP	1992–1997 [5 years]

de Mos et al. 2007 [5]	Source population 190 902 assessed from 46 general medical practices	Sensory Vasomotor Sudomotor Motor/trophic Neurologic Complaints Alternative diagnoses	Postmenopausal female gender and having a fracture; upper limb affected more frequently than the lower limb	3 sets of criteria Veldman, IASP, and Harden/Bruehl	1996–2005 [9 years]

Sandroni et al. 2003 [4]	Source population 106 470 with unified access to all patient records	Clinical characteristics Signs and symptoms Laboratory tests Response to treatment	Risks for onset of CRPS 1 were identified as female gender or suffering an upper limb fracture	IASP	1989–1999 [10 years]

Duman et al. 2007 [43]	168 males in Turkish military hospitals	Inciting injury Location Hospitalisation	Inciting event for onset of RSD was fracture in 55.3%, incisive trauma in 16.7%, and soft tissue sprains/strains in 28%	IASP and three-phase bone scan	2003–2006 [3 years]

van Rijn et al. 2007 [22]	Neurology outpatient clinic study of 185 patients with CRPS 1, 86.5% females, mean age at onset of CRPS 37.5 ± 15.4 years, 91% of whom developed dystonia	Clinical and temporal characteristics	Earlier onset of dystonia (<1 year) to be possibly related to the same mechanism and that delayed onset dystonia was related to another mechanism; 86.5% of participants were female; the inciting injury for CRPS 1 was soft tissue in 49.7%, fracture in 25.9%, and surgery in 24.3	IASP	1998–2004 [6 years]

Anderson and Fallat 1999 [44]	33 patients with lower limb CRPS 1 or sympathetically maintained pain; 60% were female; group average age 43.5 ± 12.6 years)	Clinical characteristics Type of injury or surgery Time to diagnosis Signs and symptoms Treatment	Fracture was the most common cause for injury (45%); trauma accounted for 73%	Not given other than being confirmed by an anaesthesiologist at the pain management centre	1990–1997 [7 years]

**Table 5:** Results presenting quality and relevance of data extraction for onset of CRPS 1 from prospective studies.

Prospective studies	Population sample representative	Adequate control group	Study attrition described	Risk/predictor outcome adequately defined	Risk/predictor outcome adequately measured	Analysis statistically appropriate	Quality
Harden et al. 2003 [32]	No	No	Yes	Yes	Yes	Yes	Adequate
Schürmann et al. 2000 [33]	No	No	No	Yes	Yes	No	Poor
Puchalski and Zyluk. 2005 [34]	No	No	Yes	Partly	Yes	Yes	Poor
Beerthuizen et al. 2012 [35]	Yes	Yes	Yes	Yes	Yes	Yes	Good
Dijkstra et al. 2003 [36, 46]	No	Yes	Yes	Partly	No	No	Poor
Dilek et al. 2012 [37]	No	No	Yes	Yes	Yes	No	Adequate
Jellad et al. 2014 [38]	No	Yes	Yes	Yes	No	Yes	Adequate
Goris et al. 2007 [39]	No	Yes	Yes	Yes	Yes	Yes	Good
Gradl and Schürmann 2005 [40]	Partly	No	No	Yes	Yes	No	Poor
Moseley et al. 2014 [41]	Yes	Yes	Partly	Yes	No	Yes	Adequate

**Table 6:** Results presenting quality and relevance of data extraction for onset of CRPS 1 from retrospective studies.

Retrospective studies	Population sample representative	Adequate control group	Study attrition described	Risk/predictor outcome adequately defined	Risk/predictor outcome adequately measured	Analysis statistically appropriate	Quality
Allen et al. 1999 [42]	No	No	No	Yes	Yes	Yes	Adequate
de Mos et al. 2007 [5]	Yes	Yes	No	Yes	Yes	Yes	Good
Sandroni et al. 2003 [4]	Yes	No	Yes	Yes	Yes	Yes	Good
Duman et al. 2007 [43]	No	No	No	Yes	No	No	Poor
van Rijn et al. 2007 [22]	Yes	No	No	Yes	Yes	Yes	Good
Anderson and Fallat 1999 [44]	Yes	No	No	No	Yes	Yes	Poor

**Table 7:** Results showing weighted strength against possible bias risk for prospective studies' analyses with risk rating in bold italics.

Authors	Population sample selection bias risk	Study design bias risk	Funding provision bias risk	Detection bias risk	Measurement bias risk	Weighted strength across the five factors
Harden et al. 2003 [32]	77 participants, 61.6% female, awaiting TKR in single centre setting	16 develop CRPS 1 by 1 month and 7 by 6 months after TKR Mixed within/between designs	No mention of funding or conflict of interests	All samples assessed by the same physician	Point-biserial correlations due to small sample of CRPS	
	***Yes***	***Yes***	***Yes***	***Yes***	***Yes***	***Weak***

Schürmann et al. 2000 [33]	27 participants, gender percentages not given, all with distal radial fractures in single centre setting	4 out of 27 develop CRPS 1 and two are identified as borderline	Acknowledgment is given to funder support, potential conflict of interests is not mentioned	Consensus between examiners only for oedema, reliable Doppler perfusion monitor	Regression analysis	
	***Yes***	***Yes***	***Yes***	***Yes***	***No***	***Weak***

Puchalski and Zyluk 2005 [34]	121 patients, gender percentages not given, approached with distal displaced radial fractures, the day after fixation of the fracture in single centre setting	59 refuse to participate in psychological examination; 49.5% of the sample group is available for analysis	No mention of funding or conflict of interests	Sample assessed by “we,” but clarity about authors assessment for agreement is not mentioned	A Mann-Whitney *U*-test for determining statistical relationships	
	***Yes***	***Yes***	***Yes***	***Yes***	***No***	***Weak***

Beerthuizen et al. 2012 [35]	Multicentre setting of 3 hospitals in single city, telephonic interview of 748 patients, 63.6% female, with single fracture of wrist, scaphoid, ankle, or metatarsal	596 participate (80%) and 18.1% of those who developed possible CRPS signs refused or were unable to attend	Acknowledgment is given to 2 sources of funding and neither funders are involved with design, conduct, preparation, review, or approval of the manuscript	Routine examination followed up by single experienced pain specialist clinician to confirm CRPS 1 diagnosis	A Mann-Whitney *U*-test due to skewed distribution of variables, binary logistic regression analysis using SPSS	
	***No***	***Yes***	***No***	***Yes***	***No***	***Acceptable***

Dijkstra et al. 2003 [36]	All patients, gender percentages not given, with fracture of distal radius who visit a single setting approached the day after the fracture	91 participants, 3 drop-outs; only 1 female develops CRPS 1	No funding acknowledged and hospital staff thanked for cooperation	Only researchers given as assessors for CRPS 1, no other confirmation of diagnosis	Descriptive statistics used and analysis not possible with only one CRPS 1 subject	
	***Yes***	***Yes***	***Yes***	***Yes***	***Yes***	***Weak***

Dilek et al. 2012 [37]	All patients, 64% female, presenting to single setting with fractures of distal radius asked to participate in psychological assessment 2 days after cast application	74 participants with 24 drop-outs	No conflict of interests identified and no funders acknowledged, thanks given to patients who participated	No confirmation validation given for CRPS 1 diagnosis other than fulfillment of IASP criteria	Comparative statistics described with *P* value for significance but no statistical approach described	
	***No***	***Yes***	***Yes***	***Yes***	***Yes***	***Weak***

Jellad et al. 2014 [38]	All patients, 62.2% female, presenting to single setting with closed fractures of distal radius referred by orthopaedic surgeons for rehabilitation	90 participants with zero drop-outs.	No mention of funding or conflict of interests	No confirmation validation given for CRPS 1 diagnosis other than fulfillment of Veldman criteria	Logistic regression analysis using SPSS	
	***Yes***	***Yes***	***Yes***	***Yes***	***No***	***Weak***

Goris et al. 2007 [39]	Multicentre setting of 3 hospitals in 2 cities, 83.3% female	114 participants with 6 drop-outs 1 year later	No benefit of any form is declared	Confirmation of diagnosis using 2 criteria as well as assessment by 2 investigators and blinding to results at a year's follow-up	Mann-Whitney *U*-test for paired data, Kruskal-Wallis test for nonparametric data, Gunn's multiple comparison test with regression coefficient for change of regional inflammation score	
	***No***	***No***	***No***	***No***	***No***	***Strong***

Gradl and Schürmann 2005 [40]	All consecutive patients, 60% female, who develop CRPS 1 following trauma in single centre setting	10 participants	No mention of funding or conflict of interests	Confirmation of diagnosis using Harden/Bruehl criteria by 2 independent surgeons and pain specialist investigators and blinding to results at a year's follow-up	Repeated measures every 10 days for 3 months with result of measures graphically presented but no statistical approach described	
	***Yes***	***Yes***	***Yes***	***No***	***Yes***	***Weak***

Moseley et al. 2014 [41]	1661 patients, 51.5% female presenting to multicentre (3) hospital settings with fractures of distal radius not needing surgical fixation; no city(s) mentioned	1549 participants, 21 drop-outs due to administrative error, only mentioned as percentages	No mention of funding or conflict of interests	Routine examination by single experienced pain specialist clinician to confirm CRPS 1 diagnosis made by “standard criteria” listed in an appendix	A predictive model was developed using logistic regression, likelihood ratio test, with bootstrap sampling and goodness of fit with Hosmer-Lemeshow test	
	***No***	***No***	***Yes***	***Yes***	***No***	***Acceptable***

**Table 8:** Results showing weighted strength against possible bias risk for retrospective studies' analyses with risk rating in bold italics.

Authors	Population sample selection bias risk	Study design bias risk	Funding provision bias risk	Detection bias risk	Measurement bias risk	Weighted strength
Allen et al. 1999 [42]	Medical records of all consecutive CRPS 1 patients, 70% female, who were referred to a single setting multidiscipline pain centre	134 consecutive CRPS 1 patients	No mention of funding or conflict of interests	Independent review of medical records	Result of measures listed with *P* values for significance but no statistical approach described	
	***No***	***Yes***	***Yes***	***No***	***Yes***	***Weak***

de Mos et al. 2007 [5]	Electronic medical records of one country, population gender mix not given	Estimation of CRPS 1 incidence from data of 600 000 patients; control groups are those who do not develop CRPS 1	Acknowledgment of funding provided through TREND, a Dutch government research grant; no mention of conflict of interests	Potentially overstrict retrospective application of 3 sets of criteria: Veldman, IASP, and Harden/Bruehl, to electronic records by independent reviewers with *k* statistics for agreement	Poisson distribution, logistic regression, chi-square, and Student's *t*-tests using SPSS software	
	***No***	***No***	***No***	***No***	***No***	***Strong***

Sandroni et al. 2003 [4]	Electronic medical records of one region, population gender mix not given	Estimation of CRPS 1 incidence from data of 70 745 patients	Acknowledgment of funding provided through two sources, no conflict of interests mentioned	Potentially lenient retrospective application of IASP criteria to electronic records by one reviewer with 10% random independent assessment and 93.4% agreement	Chi-square, Fisher's exact, and Wilcoxon rank sum tests with no software described	
	***No***	***No***	***No***	***Yes***	***No***	***Acceptable***

Duman et al. 2007 [43]	Medical records of two tertiary military hospitals in one country, all male	168 CRPS 1 patients	No mention of funding or conflict of interests	Author review with no independent assessment using IASP criteria and three-phase bone scan	Descriptive statistics only using SPSS	
	***Yes***	***Yes***	***Yes***	***Yes***	***Yes***	***Weak***

van Rijn et al. 2007 [22]	Medical records of all CRPS patients, 86.5% female, who were referred to a single setting movement disorder centre	121 CRPS 1 patients	Acknowledgment of funding provided through TREND, a Dutch government research grant; no mention of conflict of interests	Author review with no independent assessment using IASP criteria	A multivariate analysis using Cox's proportional hazards model, Mann-Whitney *U*-test, chi-square tests, and Student's *t*-tests using SPSS software	
	***No***	***Yes***	***No***	***Yes***	***No***	***Acceptable***

Anderson and Fallat 1999 [44]	Medical records of all CRPS patients, 61% female, seen in a single setting foot and ankle trauma clinic	33 CRPS 1 patients	No funding mentioned and acknowledgment of assistance with statistical analysis and illustrations	CRPS 1 diagnosis confirmed in records by medical specialist at pain management centre	Regression analysis, unpaired *t*-test, paired *t*-test, and Pearson's correlation using SPSS	
	***Yes***	***Yes***	***Yes***	***Yes***	***No***	***Weak***

**Table 9:** Results summary showing factors examined and not found to be risk factors for the onset of CRPS 1 with weighting bias strength and quality and relevance.

Not a risk factor for CRPS onset	Evidence source	Weighting bias strength	Quality and relevance of data extraction
Preoperative psychological distress or pain levels	Puchalski and Zyluk 2005 [34]	Weak	Poor
Diagnostic bone scan	Allen et al. 1999 [42]	Weak	Adequate
Psychological behaviour: depression	Harden et al. 2003 [32]	Weak	Adequate

**Table 10:** Results summary showing possible risk factors for the onset of CRPS 1 with weighting bias strength and quality and relevance.

Risk factors for CRPS onset	Evidence source	Weighting strength against bias	Quality and relevance of data extraction
Female gender	Puchalski and Zyluk 2005 [34]	Weak	Poor
	Dijkstra et al. 2003 [36, 46]	Weak	Poor
	Dilek et al. 2012 [37]	Weak	Adequate
	Sandroni et al. 2003 [4]	Acceptable	Good
	Allen et al. 1999 [42]	Weak	Adequate
	van Rijn et al. 2007 [22]	Acceptable	Good

Postmenopausal female gender	Beerthuizen et al. 2012 [35]	Acceptable	Good
	Jellad et al. 2014 [38]	Weak	Adequate
	de Mos et al. 2007 [5]	Strong	Good
	Sandroni et al. 2003 [4]	Acceptable	Good

Fracture of distal radius or an ankle dislocation or intra-articular fracture	Beerthuizen et al. 2012 [35]	Acceptable	Good
	Sandroni et al. 2003 [4]	Acceptable	Good
	de Mos et al. 2007 [5]	Strong	Good
	Duman et al. 2007 [43]	Weak	Poor
	Anderson and Fallat 1999 [44]	Weak	Poor

Immobilisation	Allen et al. 1999 [42]	Weak	Adequate

Report of higher than usual levels of pain in early phase of trauma	Beerthuizen et al. 2012 [35]	Acceptable	Good
	Jellad et al. 2014 [38]	Weak	Adequate
	Moseley et al. 2014 [41]	Acceptable	Adequate
